# A Novel Method for Remaining Useful Life Prediction of RF Circuits Based on the Gated Recurrent Unit–Convolutional Neural Network Model

**DOI:** 10.3390/s24092841

**Published:** 2024-04-29

**Authors:** Wanyu Yang, Kunping Wu, Bing Long, Shulin Tian

**Affiliations:** 1 School of Automation Engineering, University of Electronic Science and Technology of China (UESTC), Chengdu 611731, China; kunping_wu@163.com (K.W.); longbing@uestc.edu.cn (B.L.); shulin@uestc.edu.cn (S.T.); 2 Shenzhen Institute for Advanced Study, University of Electronic Science and Technology of China (UESTC), Shenzhen 518000, China

**Keywords:** remaining useful life (RUL), RF circuit, gated recurrent unit (GRU), convolutional neural network (CNN), reliability

## Abstract

The remaining useful life (RUL) prediction of RF circuits is an important tool for circuit reliability. Data-driven-based approaches do not require knowledge of the failure mechanism and reduce the dependence on knowledge of complex circuits, and thus can effectively realize RUL prediction. This manuscript proposes a novel RUL prediction method based on a gated recurrent unit–convolutional neural network (GRU-CNN). Firstly, the data are normalized to improve the efficiency of the algorithm; secondly, the degradation of the circuit is evaluated using the hybrid health score based on the Euclidean and Manhattan distances; then, the life cycle of the RF circuits is segmented based on the hybrid health scores; and finally, an RUL prediction is carried out for the circuits at each stage using the GRU-CNN model. The results show that the RMSE of the GRU-CNN model in the normal operation stage is only 3/5 of that of the GRU and CNN models, while the prediction uncertainty is minimized.

## 1. Introduction

Radio frequency circuits (RF circuits) refer to circuits that process signals with wavelengths that are on the same order of magnitude as the component size [1] and are widely used in the commercial, civilian, and defense fields. However, due to their complex circuit structure and working environment, it is easy to encounter problems such as circuit performance degradation and system failure [2,3,4,5,6]. When a failed RF circuit is in a personal mobile device, it can lead to poor personal communication, but when it is in a large system such as radar, a weapon, etc., it can cause a great loss of life and property.

Remaining useful life (RUL) is the amount of time a device can continue to operate safely within its expected lifespan [7,8,9]. RUL prediction is an important part of Prognostics and Health Management (PHM). It can predict the failure time of equipment before it fails and prepare for shutdown and maintenance in advance. This can reduce redundant maintenance, lower maintenance costs, and effectively reduce the risk of catastrophic accidents and associated damage, greatly improving the reliability of the system. Therefore, it is extremely important to realize a highly accurate RUL prediction for RF circuits.

Commonly used RUL prediction methods fall into three main categories: model-driven approaches, data-driven approaches, and hybrid approaches combined the data-driven approaches and model-driven approaches.

Model-driven approaches often require a comprehensive understanding of the degradation mechanism of circuits, and they describe the physicochemical phenomena generated during the degradation of the circuit by modeling. Currently, limited by the complexity of circuit structures and the high difficulty of modeling, few people have conducted in-depth analyses of the degradation mechanism of a complete circuit. Many scholars are studying the degradation mechanism of commonly used devices in circuits. Yao Bo et al. [10] studied the failure mechanism of AC filter capacitors, and the results showed that the typical precursor capacitance remained almost unchanged during the degradation process when the applied AC voltage varied within a certain range, and when the temperature rise reached 3–4 times the initial temperature rise, the AC capacitor rapidly failed within ten minutes. Due to the short failure time, it is almost impossible to realize an RUL prediction for AC filter capacitors. Yanjun Xu et al. [11] concluded that current-induced electromigration (EM), and Ni diffusion to form porous holes, is the main reason for the EM failure of NiCu thin film, and realized their life prediction based on the experimental results.

The data-driven approaches are the mainstream method for researching RUL prediction in various fields at present. Without the need to study the complex degradation mechanism, a high-precision RUL prediction can be realized as long as there is enough data. The commonly used neural networks for RUL prediction are mainly the long short-term memory (LSTM) neural network [12] and the gated recurrent unit (GRU) neural network [13]. For example, Xiaowu Chen et al. [8] proposed a degradation model based on the Wiener process, which was combined with the LSTM neural network to realize the RUL prediction of batteries. Li Biao et al. [14] combined an attention mechanism and LSTM neural network to realize the RUL prediction of rolling bearings. Bing Long et al. [13] used a GRU neural network to predict the RUL of a hydrogen–oxygen fuel cell. The LSTM neural network and GRU neural network come from the improvement of the recurrent neural network (RNN), which is a neural network specially used to deal with time series. The RNN thinks that there is a connection between each data point of the time series, and it can dig deep into the connection to realize the time series prediction with high accuracy.

Hybrid approaches are proposed in the hope of combining the advantages of both the model-driven approaches and data-driven approaches, and it is currently common to use data-driven approaches to create a state space mapped to a state space, and then use a sensor to measure the state space of the model, to model the evolutionary degradation state [15]. For example, Jouin et al. [16] proposed three empirical models, linear, logarithmic, and exponential, for the RUL prediction of fuel cells, where the parameters of the model were obtained by particle filtering, and then the parameter-updated empirical model was used to predict the aging trend of the voltage. Yu Zang et al. [17] considered the lack of sufficient life cycle data for the D-type cables of high-speed railroad transmission equipment, and the lack of failure physical models, so they obtained the data from Ansys and used it to predict the aging trend of the voltage model; so, after obtaining the life cycle data through Ansys, the RUL of D-type cables was predicted using a hybrid of particle-filtering methods and the Paris–Laws model.

Considering the above three RUL prediction methods, the data-driven method is widely used in the PHM of circuits because of its low cost and there being no need to analyze the circuit degradation mechanism. However, most of the applications are for low-frequency analog circuits, while applications in the RF field focus on fault diagnosis. Convolutional neural networks (CNNs) show great potential in fault diagnosis and feature extraction. Kasem Khalil et al. [18] implemented the early fault diagnosis of transistors based on an FFT, PCA, and CNN to detect aging, short circuit, and open circuit faults in transistors with high accuracy. Jiyuan Gao et al. [19] used the enhanced golden eagle optimizer algorithm to optimize the 1-D CNN for the analog circuit fault diagnosis of a four-op-amp biquadratic filter circuit. Xinjia Yuan et al. [20] proposed an analog fault diagnosis method based on tunable Q-factor wavelet transform (TQWT) and CNNs, using CNNs for feature extraction and fault diagnosis, which was verified on a second-order bandpass filter circuit.

However, the analysis and measurement of RF circuits is different from that of low-frequency analog circuits, and proprietary RUL prediction methods need to be established for RF circuits. In this manuscript, an RUL prediction method for RF circuits based on a data-driven approach is proposed. The research implications of this manuscript are summarized as follows:

(1) Application area: A complete methodology for solving RUL prediction for RF circuits is proposed in four parts—namely, the establishment of a feature matrix, circuit health state assessment, circuit lifecycle segmentation, and data-driven prediction methodology based on data—which contributes to the PHM of RF circuits.

(2) Prediction model: A novel RUL prediction method combined with a GRU and convolutional neural network (CNN) is proposed. Based on the spatiotemporal information of the feature matrix, the relevant temporal information is first extracted by the GRU and mapped to a 2D matrix, and then the CNN is utilized to complete the final prediction.

The structure of this manuscript is summarized below. Section 2 describes how the simulation data are acquired and the hybrid health scores used to assess the health status. Section 3 describes in detail the basic theory of CNN and GRU models and the proposed GRU-CNN method. Section 4 gives the experimental results. Section 5 summarizes all the work.

## 2. Data Acquisition and Pre-Processing

This section describes how to obtain the simulation data and gives the calculation of the hybrid health score used to assess the health state.

### 2.1. Obtaining Simulation Data

**Figure 1:**
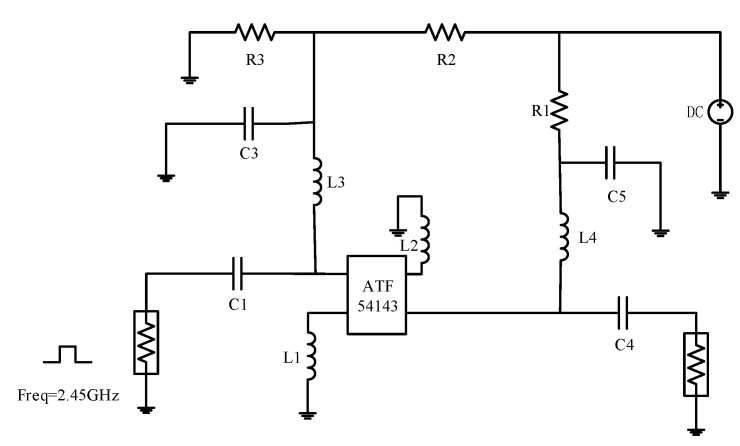
Schematic of LNA circuit based on ATF54143.

**Figure 2:**
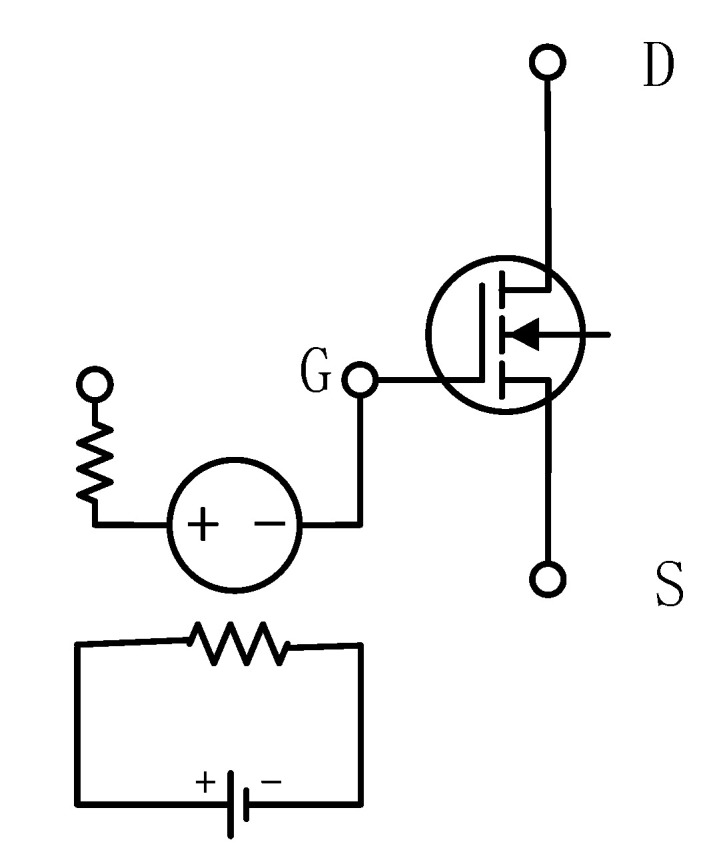
Schematic of simulating degradation of transistor.

The degradation data used in this manuscript are obtained by ADS. The schematic of the low noise amplifier circuit based on ATF54143 is shown in Figure 1. The center frequency of the circuit is 2.45 GHz and the operating frequency range is 2.40–2.50 GHz; the main devices are capacitors, resistors, inductors, and the ATF54143 low noise amplifier. Active devices can be more susceptible to environmental and wiring effects that can cause degradation, compared to passive devices. Therefore, the degradation of this RF circuit is due to the degradation of the ATF54143. In this manuscript, the degradation of the transistor due to hot carrier injection is simulated by connecting a voltage-controlled voltage source in series in front of the transistor of the ATF54143 [21], as shown in Figure 2. Where G, D, S are the gate, drain and source of the transistor respectively.

Considering the common measurement indexes of RF circuits, the S-parameter, input VSWR, output VSWR, stability, and noise figure are selected as the feature parameters. Their main definitions and expressions are shown in Table 1.

According to the common measurement indexes and operating frequency range of RF circuits, a feature matrix $F^{t}$ is established to characterize the circuit, and the feature matrix $F^{55}$ at t=55 is shown in Table 2. The rows of the feature matrix represent different frequency values, and the columns represent the feature parameters, including the four S-parameters, input/output VSWR, stability, and noise figure.

### 2.2. Data Normalization

The input feature matrix is to be normalized by columns to limit the data to a specific range, which can process the data more effectively and quickly and improve the efficiency of the algorithm. The formula for data normalization is shown below:

(1) $$x^{\prime }=\frac{x-x_{min}}{x_{max}-x_{min}}$$

where $x^{\prime }$ is the normalized value scaled to the range of [0,1] by (1). x is the data to be processed and $x_{max}$ and $x_{min}$ are the maximum and minimum values of the feature parameter, respectively. The normalized feature matrix ${F^{55}}^{\prime }$ at t=55 is shown in Table 3.

### 2.3. Hybrid Health Score

The feature parameters of the RF circuit will be degraded with different trends, so it is necessary to set the health score to integrate the degradation trajectories of the eight feature parameters. A good health score can show the health state of the RF circuit in a more intuitive form. In this manuscript, we use the hybrid distance to calculate the health score of the circuit at time t as:

(2) $$H_{t}=g({F^{t}}^{\prime },{F^{0}}^{\prime })$$

where $g({F^{t}}^{\prime },{F^{0}}^{\prime })$ is calculated based on the Manhattan distance and Euclidean distance as shown in (3). $g({F^{t}}^{\prime },{F^{0}}^{\prime })$ denotes the distance between the normalized feature matrix of the RF circuit at the moment t and the moment 0.

(3) $$g\left({F^{t}}^{\prime },{F^{0}}^{\prime }\right)=\frac{1}{2}[M\left({F^{t}}^{\prime },{F^{0}}^{\prime }\right)+E({F^{t}}^{\prime },{F^{0}}^{\prime })]$$

The formula for calculating the Manhattan distance is shown in (4).

(4) $$M\left({F^{t}}^{\prime },{F^{0}}^{\prime }\right)=\sum_{i=0}^{10}\sum_{j=0}^{7}\left|{{F^{t}}^{\prime }}_{ij}-{{F^{0}}^{\prime }}_{ij}\right|$$

The formula for the Euclidean distance is as follows:

(5) $$E\left({F^{t}}^{\prime },{F^{0}}^{\prime }\right)=\sqrt{\sum_{i=0}^{10}\sum_{j=0}^{7}{\left({{F^{t}}^{\prime }}_{ij}-{{F^{0}}^{\prime }}_{ij}\right)}^{2}}$$

## 3. Methodology

This section begins with a brief description of CNN and GRU models, then elaborates on the GRU-CNN model proposed in this manuscript for the phased RUL prediction of RF circuits, and concludes with a description of the metrics used for model performance evaluation.

### 3.1. CNN

A convolutional neural network (CNN) is an algorithm based on convolutional computation for simulating biological neural networks. A CNN’s feature learning, parameter sharing, spatial localization, and other points make it advantageous in processing two-dimensional data. Generally speaking, a CNN can be split into Convolutional Layers, Pooling Layers, and Fully Connected Layers. The Convolutional Layer is responsible for extracting data features through convolutional computation; the Pooling Layer is responsible for processing the data in the Convolutional Layer and down-sampling the data to retain the salient features; and the Fully Connected Layer is responsible for categorizing the features obtained in the previous processing.

**Figure 3:**
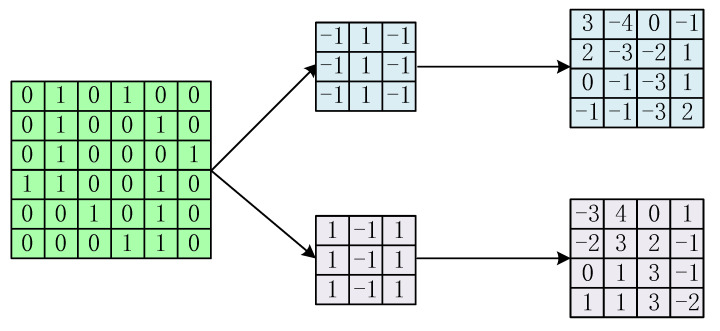
Convolutional operation of CNN.

The convolution operation of a CNN is shown in Figure 3. For a matrix to be processed, a convolution kernel is placed on the matrix and then slid over it, so that the convolution kernel matches and multiplies with a specific part of that matrix. This process can be realized by matrix multiplication, and the output of this convolution operation results in a new feature mapping. With different convolution kernels, it is possible to map the original matrix to a different feature space, and by sliding the convolution kernel, it is possible to find the relationship between the different inputs in a 2D matrix.

### 3.2. GRU

Compared to other neural networks, a recurrent neural network (RNN) has the ability to memorize, and it introduces a ring structure to ensure that the characteristics of the data can be stored for a long period and linked to the current task. This feature makes it widely used in the processing of time series data. Traditional RNN networks cannot run for a long time due to gradient explosion and gradient vanishing. Based on RNNs, long short-term memory (LSTM) and gated recurrent units (GRUs) have been proposed. Both of these can solve the problems of the gradient explosion and gradient vanishing of traditional RNN networks.

**Figure 4:**
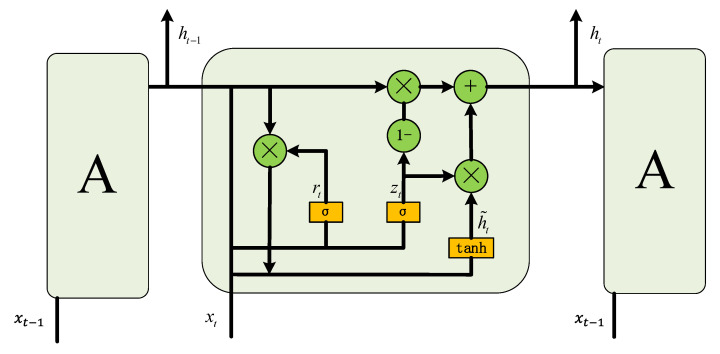
Cell structure of GRU.

LSTM innovatively introduces the function of forgetting, which can memorize part of the information and choose to forget part of the information, to ensure that the memorized information is not redundant, thus improving the problem that traditional RNN networks cannot process data for a long period [22]. LSTM uses three gates to realize this function, which are the input gate, forgetting gate, and output gate. Compared to LSTM, a GRU optimizes the structure of LSTM’s single unit of three gates into two gates, i.e., combining the output gate and the forgetting gate into an update gate, and changing the input gate into a reset gate. A GRU has a much simpler structure, and has a much higher efficiency in achieving the same effect, as LSTM. The structure of a GRU is shown in Figure 4.

The computational process of each unit of a GRU neural network is shown below. First, the two most important structures in the GRU neural network, the gating signals, reset gate $r_{t}$, and update gate $z_{t}$, are obtained from the input of the hidden layer at the current moment and the output of the hidden layer at the previous moment. These two gating signals are obtained from the output of the hidden layer at the previous moment and the input of the current moment. These two gating signals are the source of the gate in the GRU neural network. The reset gate exists to realize the memory function; when the inputs of this unit (including the inputs of the current moment and the outputs of the hidden layer of the previous moment) arrive, firstly, the reset gate is used to save these data, i.e., (8). Tanh() exists to realize the nonlinearity. Next, the data are forgotten and output using the update gate; $\left(1-z_{t}\right)$ in (9) is selective forgetting, and the $z_{t}$ part is selective memorization. Through the two stages of forgetting and remembering, the amount of memory kept in the GRU neural network is constant; thus, gradient vanishing and gradient explosion can be avoided.

(6) $$r_{t}=\sigma (W_{r}\cdot [h_{t-1},x_{t}])$$

(7) $$z_{t}=\sigma (W_{z}\cdot [h_{t-1},x_{t}])$$

(8) $${\tilde{h}}_{t}=tanh(W\cdot [r_{t}*h_{t-1},x_{t}])$$

(9) $$h_{t}=\left(1-z_{t}\right)*h_{t-1}+z_{t}*{\tilde{h}}_{t}$$

### 3.3. GRU-CNN

**Figure 5:**
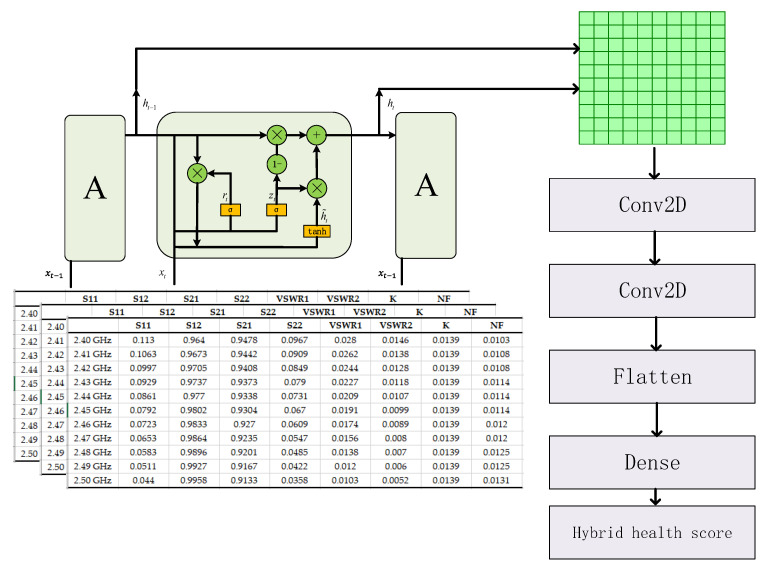
GRU-CNN model for RUL prediction of RF circuits.

The GRU-CNN model for the RUL prediction of RF circuits proposed in this manuscript is shown in Figure 5. The prediction length is L. The feature matrices of L moments are first input into the GRU neural network, which utilizes the advantage of the GRU neural network in time series processing to extract the time-related features and map them to the high-dimensional space. The output of the hidden layer in the GRU neural network is then extracted and combined into an n×n two-dimensional matrix. This 2D matrix is next passed through two Convolutional Layers, a Flatten Layer and a Dense Layer, to finally output the hybrid health score for the next moment. The method makes a mapping between the feature matrix at L moments and the hybrid health score at the next moment, integrating a large amount of feature extraction and prediction work together in this GRU-CNN model.

### 3.4. Evaluation Metrics

The evaluation metrics of the model are used to assess the generalization ability of the model; in this manuscript, we use the RMSE (Root Mean Square Error), MAPE (Mean Absolute Percentage Error), and $R^{2}$ (Coefficient of Determination) to evaluate the model. The formulas for these three metrics are shown below:

(10) $$RMSE=\sqrt{\frac{1}{m}\sum_{t=1}^{m}{\left(h_{t}-{\hat{h}}_{t}\right)}^{2}}$$

(11) $$MAPE=\frac{1}{m}\sum_{t=1}^{m}\left|\frac{{\hat{h}}_{t}-h_{t}}{h_{t}}\right|\times 100\%$$

(12) $$R^{2}=1-\frac{\sum_{t=1}^{m}{\left(h_{t}-{\hat{h}}_{t}\right)}^{2}}{\sum_{t=1}^{m}{\left({\hat{h}}_{t}-h_{t}\right)}^{2}}$$

where $h_{t}$ is the true hybrid health score at moment t and ${\hat{h}}_{t}$ is the predicted hybrid health score at moment t. The RMSE indicates the degree of dispersion of the estimation error from the mean, the MAPE is used to evaluate the degree of deviation between the true value and the fitted value, and the $R^{2}$ is a statistic that measures the goodness-of-fit of the model, which indicates the difficulty of fitting the regression model to the observations. These three evaluation metrics all evaluate the model for the accuracy of the model.

However, the high precision of the model cannot indicate the stability of the model’s prediction results. Therefore, in this manuscript, the reliability of the model is evaluated by the uncertainty of the model calculated by the deep ensemble method [23], with the formula shown in (13).

(13) $$predictive\;uncertainty=-\frac{1}{m}\sum_{t=1}^{m}logp_{\theta }\left(h_{t}|{\hat{h}}_{t}\right)$$

where,

(14) $$logp_{\theta }\left(h_{t}|{\hat{h}}_{t}\right)=-\frac{log\sigma_{\theta }^{2}({\hat{h}}_{t})}{2}-\frac{{\left(h_{t}-\mu_{\theta }({\hat{h}}_{t})\right)}^{2}}{2\sigma_{\theta }^{2}({\hat{h}}_{t})}$$

## 4. Results and Discussion

The experimental equipment information is as follows: the processor of the computer is Intel i7-9300H @ 2.40 GHz, the onboard RAM is 8 GB, and the programming environment is Python 3.8.0. This section proposes a methodology for segmenting the life cycle of RF circuits and shows the prediction results of GRU-CNN models for the normal operation phase and the slow degradation phase of RF circuits.

### 4.1. Health Score

**Figure 6:**
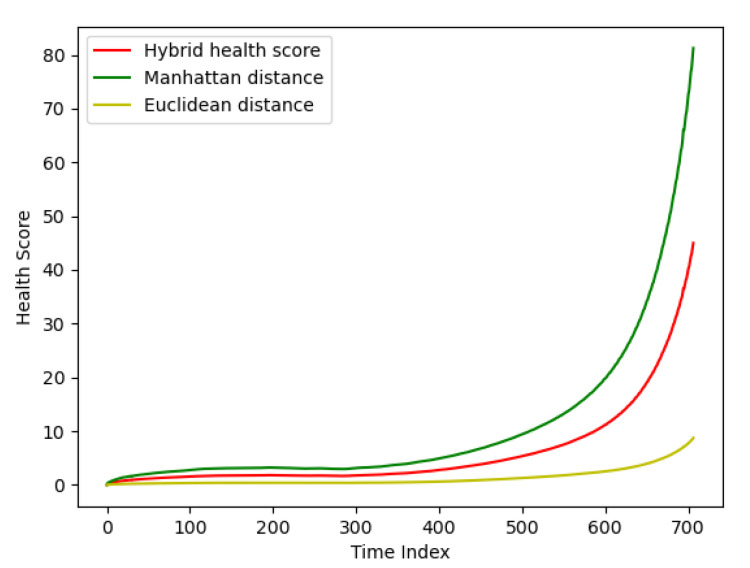
Comparison of three distance scores.

This section demonstrates the comparison results of the hybrid health scores based on Euclidean distance and Manhattan distance, health scores based on Euclidean distance, and health scores based on Manhattan distance, as shown in Figure 6, where the *x*-axis represents the different sampling moments and the *y*-axis is the health scores.

Table 4 demonstrates Pearson’s correlation coefficients between the three distance scores and the RF circuit feature parameters. It can be seen that the hybrid distance scores have improved Pearson’s correlation coefficients for five feature parameters, compared to the health scores based on the Euclidean distance. And compared with the health score based on the Manhattan distance, the range of its health score is restricted, which can improve the accuracy of prediction.

### 4.2. RF Circuit Life Cycle Segmentation

**Figure 7:**
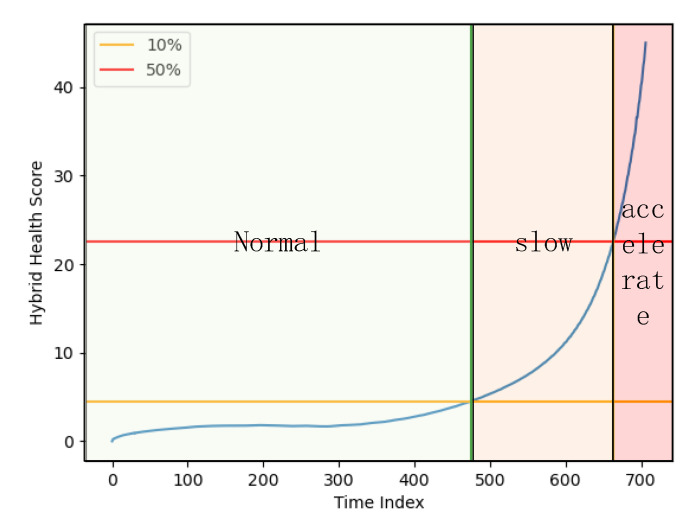
RF circuit degradation trends and life cycle segmentation.

The degradation trend of the life cycle for RF circuits is not static, and Figure 7 shows the degradation trajectory (blue line) of an RF circuit drawn from the hybrid health scores in Section 2.3. From the figure, it can be seen that, in the life cycle of RF circuits, they first experience a relatively smooth period of normal working, and then, after reaching a certain stage, their performance starts to degrade slowly, and then they will enter an accelerated degradation stage. In this manuscript, the hybrid health score of the RF circuit will be divided into three stages of its entire life cycle. When the RF circuit’s hybrid health score for the change is less than 10% of the entire life cycle of the change in health score, the circuit is considered to be in the normal working of the stage (green area). When the RF circuit’s hybrid health score for the change is 10–50% of the entire life cycle of the change in the health score, it is considered that the circuit is in the slow degradation stage (orange area). If the change of the hybrid health score of the RF circuit is greater than 50% of the change in the health score of the entire life cycle, the circuit has completely failed to complete its work and enters the accelerated degradation stage (red area). Therefore, the main stages that need to be predicted are the first two, the normal working stage and the slow degradation stage.

### 4.3. The Prediction Result of the Normal Working Stage

**Figure 8:**
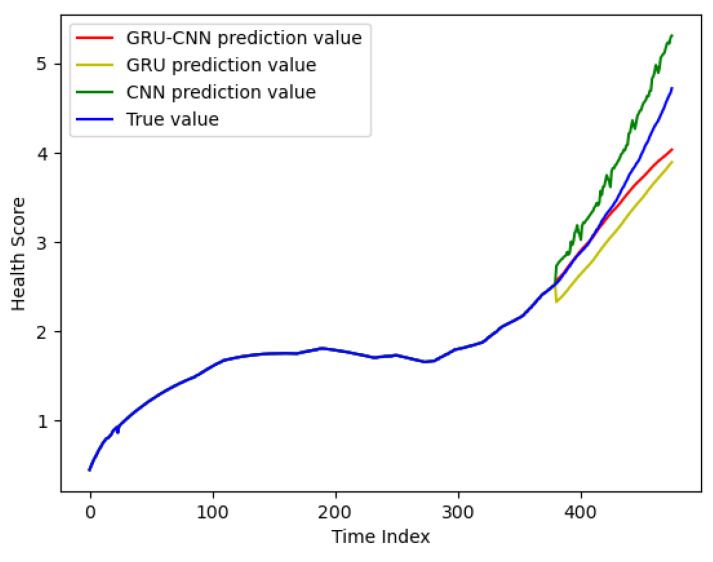
The prediction results for the normal working stage.

Figure 8 illustrates the experimental results in the normal working stage, with a prediction starting point of 380. There are four curves in the figure, where the blue curve is the real degradation curve, the red curve is the prediction curve of the GRU-CNN model proposed in this manuscript, the green curve is the prediction curve of the CNN model, and the yellow curve is the prediction curve of the GRU model. From the figure, it can be seen that the prediction curve of the GRU-CNN model is closest to the real degradation curve, and the prediction results in the early stage almost coincide with the real degradation curve. In the later stage, as the prediction time becomes longer, the error gets bigger and bigger.

The comparative results of the quantization of the three models are summarized in Table 5. The GRU-CNN model has the smallest RMSE and MAPE, which proves that the model has the highest prediction accuracy in the normal operation phase of RF circuits. The *R*^2^ = 0.9775 also indicates that the GRU-CNN model has the best fitting effect. At the same time, the GRU-CNN model also has the lowest prediction uncertainty, which indicates the high reliability of the prediction accuracy of the model. Compared with the GRU-CNN model, the GRU model neglects the spatial connection of the feature matrix in the prediction, and predicts only through the temporal connection of the feature matrix; while the CNN model neglects the sequential information of the feature matrix, and predicts only through the spatial characteristics, which results in the accuracy of the prediction methods of both the GRU model and the CNN model being slightly lower than that of the GRU-CNN model proposed in this manuscript. However, the GRU-CNN model has the longest prediction time, because it needs to extract the features in both the temporal and spatial parts. The high accuracy and low prediction uncertainty of the GRU-CNN model’s prediction is enough to compensate for its shortcomings in prediction time.

### 4.4. The Prediction Result of the Slow Degradation Stage

**Figure 9:**
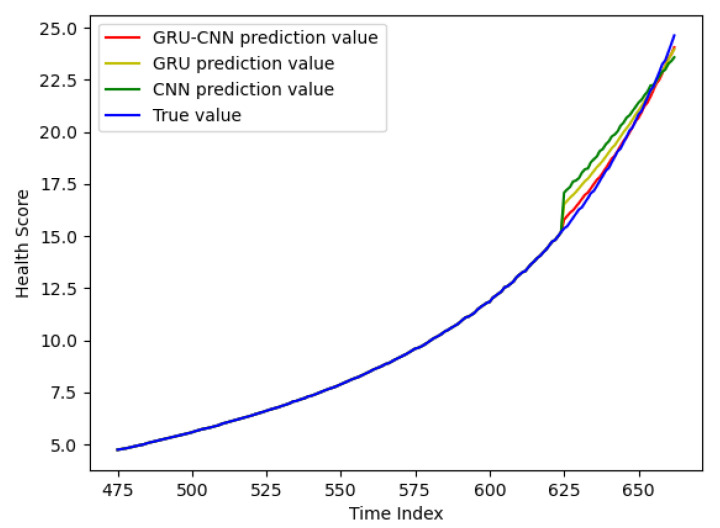
The prediction results for the slow degradation stage.

Figure 9 shows the prediction results for RF circuits in the slow degradation stage. The same as Figure 8, there are four curves, blue (real degradation data), red (GRU-CNN model), yellow (GRU model), and green (CNN model). Again, the red curve and the blue curve almost overlap, indicating that the GRU-CNN model has the highest prediction accuracy. When the RF circuit enters the accelerated degradation stage, the circuit cannot work. It is considered to be the end of life of the RF circuit. As can be seen in Figure 9, the GRU-CNN model’s prediction result at the last moment is closest to the real degradation data, so its accuracy in judging the end of life is much higher than that of the GRU model and the CNN model.

Table 6 demonstrates an evaluation of the prediction results of the GRU-CNN model, the GRU model, and the CNN model in the slow degradation stage. It can be seen that the RMSE of the GRU-CNN model is only 0.1283, which is 40% that of the GRU model and 25% that of the CNN model, and the MAPE is also the lowest, 40% that of the GRU model and 24% that of the CNN model. Meanwhile, the GRU-CNN model has the highest *R*^2^, indicating that the model fits the data best. Similarly, it has the lowest prediction uncertainty of 0.7499, indicating the highest reliability of the prediction results. In Table 5, the CNN model has the highest prediction uncertainty of 32.6022, indicating that its prediction results are not reliable.

### 4.5. Discussion

Based on the analysis of the results in Section 4.3 and Section 4.4, it can be found that the GRU-CNN model has the highest prediction accuracy, the best fitting effect, and the lowest prediction uncertainty. Therefore, among the three models compared in this manuscript, GRU-CNN is the best solution for the RUL prediction of RF circuits. By comparing the prediction results of the three models, it can be found that the GRU model can extract the sequence features of the input series and store the previous calculations as “memory” in the network, so it can ensure that the prediction results of the model will fluctuate within a small range, whereas the CNN model does not extract information in the time dimension, so it may have a larger error, leading to extremely high prediction uncertainty. The CNN model can extract the information on space that is ignored by the GRU model, thus improving the prediction accuracy of the GRU model.

The proposed RUL prediction method for RF circuits has four main parts, a feature matrix, circuit health assessment, circuit lifecycle segmentation, and data-driven prediction method with GRU-CNN. Although the method is validated with only one low-noise amplified circuit, the methods of circuit health assessment, circuit lifecycle segmentation, and GRU-CNN are generalized. For different kinds of RF circuits, different feature parameters need to be selected to build the feature matrix with the circuit characteristics. For example, mixers must consider conversion loss, isolation, a 1 dB compression point, third-order intermodulation, etc. Power amplifiers also need to consider efficiency and distortion.

## 5. Conclusions

In this manuscript, a novel RUL prediction method for RF circuits based on the GRU-CNN model is proposed. Firstly, the data are normalized to limit all the data to the range of 0–1, which improves the processing efficiency of the model. Secondly, the degradation trends of eight feature parameters of RF circuits at 11 frequencies are integrated, using the hybrid health scores calculated based on the Manhattan distance and Euclidean distance. Then the life cycle of RF circuits is segmented into the normal working stage, slow degradation stage, and accelerated degradation stage based on the hybrid health scores. Finally, after reasonably analyzing the life cycle of RF circuits, the normal operation stage and slow degradation stage are selected for applying the GRU-CNN model proposed in this manuscript for prediction. Compared with the GRU model and CNN model, our proposed GRU-CNN model is more advantageous. The following conclusions can be drawn:

(1) The GRU-CNN model utilizes the GRU model to extract the features of the data in time, and then the CNN is used to process the features in space, which makes full use of the spatiotemporal dependence of the data. The prediction accuracies in both the normal working stage and the slow degradation stage of the RF circuit are higher than those of the GRU model and the CNN model.

(2) The temporal features extracted by the GRU model can constrain the prediction results within a range, avoiding prediction results with particularly large errors. Therefore, the prediction uncertainty of the GRU-CNN model in the normal working stage and the slow degradation stage of the RF circuit is much smaller than that of the CNN model.

## Figures and Tables

**Table 1:** Introduction to feature parameters.

Feature Parameters	Definition	Formulas
S-parameter	Used to describe the transmission and reflection characteristics of radio frequency energy in multi-port networks.	$S=\left[\begin{array}{cc} S_{11} & S_{12}\\ S_{21} & S_{22} \end{array}\right]=\left[\begin{array}{cc} \frac{b_{1}}{a_{1}} & \frac{b_{1}}{a_{2}}\\ \frac{b_{2}}{a_{1}} & \frac{b_{2}}{a_{2}} \end{array}\right]$
VSWR	Used to indicate the matching of input and output circuits.	$VSWR=\frac{1-\left|\Gamma_{1}\right|}{1+\left|\Gamma_{1}\right|}$
Stability	Reflects the ability of a circuit to maintain normal operation in the face of environmental changes.	$K=\frac{1-{\left|S_{11}\right|}^{2}-{\left|S_{22}\right|}^{2}+{\left|S_{11}S_{22}-S_{21}S_{12}\right|}^{2}}{2\left|S_{12}S_{21}\right|}$
Noise figure	Indicates the signal-to-noise ratio reduction factor.	$NF\left(dB\right)=10\mathrm{lg}\left(NF\right)=10lg(\frac{S_{in}/N_{in}}{S_{out}/N_{out}})$

**Table 2:** Feature matrix $F^{55}$ at t=55.

	S11	S12	S21	S22	VSWR1	VSWR2	K	NF
2.40 GHz	−14.172	−13.422	12.419	−14.988	1.486	1.433	1.003	0.192
2.41 GHz	−14.247	−13.386	12.387	−15.066	1.481	1.429	1.003	0.193
2.42 GHz	−14.322	−13.351	12.356	−15.146	1.476	1.424	1.003	0.193
2.43 GHz	−14.398	−13.316	12.324	−15.226	1.471	1.419	1.003	0.194
2.44 GHz	−14.475	−13.281	12.293	−15.306	1.466	1.414	1.003	0.194
2.45 GHz	−14.552	−13.246	12.262	−15.388	1.461	1.41	1.003	0.194
2.46 GHz	−14.63	−13.212	12.231	−15.47	1.456	1.405	1.003	0.195
2.47 GHz	−14.709	−13.178	12.2	−15.553	1.451	1.401	1.003	0.195
2.48 GHz	−14.788	−13.143	12.169	−15.637	1.446	1.396	1.003	0.196
2.49 GHz	−14.869	−13.109	12.138	−15.722	1.441	1.391	1.003	0.196
2.50 GHz	−14.949	−13.076	12.108	−15.808	1.436	1.387	1.003	0.197

**Table 3:** Normalized feature matrix ${F^{55}}^{\prime }$ at t=55.

	S11	S12	S21	S22	VSWR1	VSWR2	K	NF
2.40 GHz	0.1130	0.9640	0.9478	0.0967	0.0280	0.0146	0.0139	0.0103
2.41 GHz	0.1063	0.9673	0.9442	0.0909	0.0262	0.0138	0.0139	0.0108
2.42 GHz	0.0997	0.9705	0.9408	0.0849	0.0244	0.0128	0.0139	0.0108
2.43 GHz	0.0929	0.9737	0.9373	0.0790	0.0227	0.0118	0.0139	0.0114
2.44 GHz	0.0861	0.9770	0.9338	0.0731	0.0209	0.0107	0.0139	0.0114
2.45 GHz	0.0792	0.9802	0.9304	0.0670	0.0191	0.0099	0.0139	0.0114
2.46 GHz	0.0723	0.9833	0.9270	0.0609	0.0174	0.0089	0.0139	0.0120
2.47 GHz	0.0653	0.9864	0.9235	0.0547	0.0156	0.0080	0.0139	0.0120
2.48 GHz	0.0583	0.9896	0.9201	0.0485	0.0138	0.0070	0.0139	0.0125
2.49 GHz	0.0511	0.9927	0.9167	0.0422	0.0120	0.0060	0.0139	0.0125
2.50 GHz	0.0440	0.9958	0.9133	0.0358	0.0103	0.0052	0.0139	0.0131

**Table 4:** Pearson’s correlation coefficient for three distance scores.

	S11	S12	S21	S22	VSWR1	VSWR2	K	NF
Hybrid	0.954	−0.975	−0.949	0.949	0.901	0.988	0.990	0.991
Manhattan	0.956	−0.973	−0.951	0.947	0.903	0.989	0.989	0.991
Euclidean	0.934	−0.986	−0.928	0.967	0.874	0.978	0.994	0.987

**Table 5:** Comparison of experimental results in normal working stage.

	RMSE	MAPE	*R* ^2^	Predict Time	Predictive Uncertainty
GRU-CNN	0.1165	0.0093	0.9775	10.1734	0.4119
GRU	0.1948	0.0248	0.9225	9.6104	1.0747
CNN	0.1870	0.0195	0.9661	4.7357	4.6596

**Table 6:** Comparison of experimental results in slow degradation stage.

	RMSE	MAPE	*R* ^2^	Predict Time	Predictive Uncertainty
GRU-CNN	0.1283	0.0026	0.9994	5.4227	0.7499
GRU	0.3143	0.0065	0.9965	6.0814	1.0151
CNN	0.5111	0.0107	0.9911	3.7190	32.6022

## Data Availability

The original contributions presented in the study are included in the article, further inquiries can be directed to the corresponding author.

## References

[1] Chang D., Kitchen J.N., Kiaei S., Ozev S. (2020). In-Field Recovery of RF Circuits from Wearout Based Performance Degradation. IEEE Trans. Emerg. Top. Comput..

[2] Dermentzoglou L.E., Arapoyanni A., Tsiatouhas Y. (2010). A Built-In-Test Circuit for RF Differential Low Noise Amplifiers. IEEE Trans. Circuits Syst. I Regul. Pap..

[3] Girard M., Dubois T., Hoffmann P., Duchamp G. (2018). Effects of HPEM stress on GaAs low-noise amplifier from circuit to component scale. Microelectron. Reliab..

[4] Huang K., Stratigopoulos H.-G., Mir S. Bayesian Fault Diagnosis of RF Circuits Using Nonparametric Density Estimation. Proceedings of the 2010 19th IEEE Asian Test Symposium.

[5] Nel H.P., Dualibe F.C., Stander T. (2023). Influence of PVT Variation and Threshold Selection on OBT and OBIST Fault Detection in RFCMOS Amplifiers. IEEE Open J. Circuits Syst..

[6] Tang X., Liu Z., Liang J., Wu K., Bu Z., Chen L. A Fast Fault Diagnosis Method for RF Front-End Modules Based on Adaptive Signal Decomposition and Deep Neural Network. Proceedings of the 2023 IEEE Autotestcon 2023.

[7] Meng L., Chen Y., Zhou Z. (2022). Segmental Degradation RUL Prediction of IGBT Based on Combinatorial Prediction Algorithms. IEEE Access.

[8] Chen X., Liu Z. (2022). A long short-term memory neural network based Wiener process model for remaining useful life prediction. Reliab. Eng. Syst. Saf..

[9] Kong Z., Jin X., Xu Z., Zhang B. (2022). Spatio-Temporal Fusion Attention: A Novel Approach for Remaining Useful Life Prediction Based on Graph Neural Network. IEEE Trans. Instrum. Meas..

[10] Yao B., Zhang Y., Correia P., Wu R., Wang H. Accelerated degradation testing and failure phenomenon of metalized film capacitors for AC filtering. Proceedings of the 2023 IEEE Applied Power Electronics Conference and Exposition (APEC).

[11] Xu Y., Huang L., Chen G., Wu F., Xia W., Liu H. Electromigration—Induced failure mechanism and lifetime prediction in NiCu thin film. Proceedings of the 2014 15th International Conference on Electronic Packaging Technology.

[12] Cao Y., Gui L. Multi-Step wind power forecasting model Using LSTM networks, Similar Time Series and LightGBM. Proceedings of the 2018 5th International Conference on Systems and Informatics (ICSAI).

[13] Long B., Wu K., Li P., Li M. (2022). A Novel Remaining Useful Life Prediction Method for Hydrogen Fuel Cells Based on the Gated Recurrent Unit Neural Network. Appl. Sci..

[14] Li B., Tang B., Deng L., Zhao M. (2021). Self-Attention ConvLSTM and Its Application in RUL Prediction of Rolling Bearings. IEEE Trans. Instrum. Meas..

[15] Office J.E., Gebraeel N., Lei Y., Li N., Si X., Zio E. (2023). Prognostics and Remaining Useful Life Prediction of Machinery: Advances, Opportunities and Challenges. J. Dyn. Monit. Diagn..

[16] Jouin M., Gouriveau R., Hissel D., Péra M.-C., Zerhouni N. (2014). Prognostics of PEM fuel cell in a particle filtering framework. Int. J. Hydrogen Energy.

[17] Zang Y., Shangguan W., Cai B., Wang H., Pecht M.G. (2021). Hybrid remaining useful life prediction method. A case study on railway D-cables. Reliab. Eng. Syst. Saf..

[18] Khalil K., Eldash O., Kumar A., Bayoumi M. (2020). Machine Learning-Based Approach for Hardware Faults Prediction. IEEE Trans. Circuits Syst. Regul. Pap..

[19] Gao J., Guo J., Yuan F., Yi T., Zhang F., Shi Y., Li Z., Ke Y., Meng Y. (2024). An Exploration into the Fault Diagnosis of Analog Circuits Using Enhanced Golden Eagle Optimized 1D-Convolutional Neural Network (CNN) with a Time-Frequency Domain Input and Attention Mechanism. Sensors.

[20] Yuan X., Sheng Y., Zhuang X., Yin J., Yang S. (2024). A novel fault diagnosis method for second-order bandpass filter circuit based on TQWT-CNN: PLoS ONE. PLoS ONE.

[21] Cheney D.J., Douglas E.A., Liu L., Lo C.F., Gila B.P., Ren F., Pearton S.J. (2012). Degradation Mechanisms for GaN and GaAs High Speed Transistors. Materials.

[22] Long B., Li X., Gao X., Liu Z. (2019). Prognostics Comparison of Lithium-Ion Battery Based on the Shallow and Deep Neural Networks Model. Energies.

[23] Lakshminarayanan B., Pritzel A., Blundell C. Simple and scalable predictive uncertainty estimation using deep ensembles. Proceedings of the 31st International Conference on Neural Information Processing Systems.

